# A High-Throughput Method Based on Microculture Technology for Screening of High-Yield Strains of Tylosin-Producing *Streptomyces fradiae*

**DOI:** 10.4014/jmb.2210.10023

**Published:** 2023-03-10

**Authors:** Zhiming Yao, Jingyan Fan, Jun Dai, Chen Yu, Han Zeng, Qingzhi Li, Wei Hu, Chaoyue Yan, Meilin Hao, Haotian Li, Shuo Li, Jie Liu, Qi Huang, Lu Li, Rui Zhou

**Affiliations:** 1 National Key Laboratory of Agricultural Microbiology, College of Veterinary Medicine, Huazhong Agricultural University, Wuhan 430070, P.R. China; 2 Cooperative Innovation Center of Sustainable Pig Production, Wuhan 430070, P.R. China; 3 International Research Center for Animal Disease (Ministry of Science & Technology of China), Wuhan 430070, P.R. China; 4 Hubei Provincial Bioengineering Technology Research Center for Animal Health Products, Yingcheng 432400, P.R. China; 5 The HZAU-HVSEN Research Institute, Wuhan 430042, P.R. China

**Keywords:** Tylosin, *Streptomyces fradiae*, high-throughput screening, mutagenesis, high-yield strains

## Abstract

Tylosin is a potent veterinary macrolide antibiotic produced by the fermentation of *Streptomyces fradiae*; however, it is necessary to modify *S. fradiae* strains to improve tylosin production. In this study, we established a high-throughput, 24-well plate screening method for identifying *S. fradiae* strains that produce increased yields of tylosin. Additionally, we constructed mutant libraries of *S. fradiae* via ultraviolet (UV) irradiation and/or sodium nitrite mutagenesis. A primary screening of the libraries in 24-well plates and UV spectrophotometry identified *S. fradiae* mutants producing increased yields of tylosin. Mutants with tylosin yield 10% higher than the wild-type strain were inoculated into shake flasks, and the tylosin concentrations produced were determined by high-performance liquid chromatography (HPLC). Joint (UV irradiation and sodium nitrite) mutagenesis resulted in higher yields of mutants with enhanced tylosin production. Finally, 10 mutants showing higher tylosin yield were re-screened in shake flasks. The yield of tylosin A by strains UN-C183 (6767.64 ± 82.43 μg/ml) and UN-C137 (6889.72 ± 70.25 μg/ml) was significantly higher than that of the wild-type strain (6617.99 ± 22.67 μg/ml). These mutant strains will form the basis for further strain breeding in tylosin production.

## Introduction

Tylosin is a veterinary antibiotic mainly used for treatment of bacteria and mycoplasma infections. It is a 16-membered ring macrolide antibiotic consisting of a tylactone and three deoxyhexose sugars [1]. Tylosin is mainly produced by *Streptomyces fradiae* [2], *Streptomyces rimosus*, and *Streptomyces hygroscopicus* [3], with *S. fradiae* being the preferred species for industrial production. Tylosin is also the raw material for the synthesis of macrolide antibiotics such as tilmicosin and tilvalsin; hence it plays a key role in the macrolide antibiotics market. However, the low yield of tylosin from *S. fradiae* limits its industrial production [4], and methods to increase this yield are considered an urgent priority for the veterinary drug industry.

To obtain high-yield, tylosin-producing strains, it is necessary to establish a high-throughput method for screening target strains from *S. fradiae* mutant libraries. In the process of fermentation, the products of *S. fradiae* mainly include four bioactive components: tylosin A, desmycosin B, macrocin C, and relomycin D [5]. In the last two steps of tylosin synthesis, demethylmacrocin O-methyltransferase catalyzes the conversion of demethylmacrocin to macrocin C. Then, under the action of TylF, O-methylation occurs on the 3’’’-hydroxyl of macrocin C, which is converted to tylosin A [6]. Tylosin A is converted to relomycin D under the effect of tylosin reductase, which changes the C-20 formyl group of tylosin A to a hydroxymethyl [7]. Desmycosin B is converted from demethyllactenocin mediated by TylE and TylF [3]. Tylosin A is the main active ingredient. The traditional screening method used to obtain high-yielding strains is culture of *S. fradiae* in shake flasks [8]. However, shake-flask screening is time-consuming, laborious, and wasteful of manpower and material resources [9]. Thus, it is unsuitable for the screening of a large number of mutants where many samples need to be analyzed. Quantitative determination of antibiotics yields is normally performed by high-performance liquid chromatography (HPLC), a technique that is sub-optimal when a large number of samples need to be analyzed. Therefore, for high-throughput screening, an alternative quantitative method is required, such as determination of tylosin yield by UV spectrophotometry [10]. High-throughput screening is also facilitated by the use of microscale methods. In recent years, 48-, 96-, or 384-well plates have been widely used for fermentation culture of microorganisms, and since *Streptomyces* require sufficient oxygen during growth, 24- and 48-well plates are the format of choice for fermentation culture [11]. As microreactors, 24- or 48-well plates allow for independent air exchange with the outside world in each well. Additionally, a “sandwich plate cover” can facilitate growth of *Streptomyces* in the wells. This type of cover consists of three layers: a stainless steel outer layer with 48 holes; a middle layer containing a filter membrane; and a silicone pad as the inner layer [12]. The plate cover can be autoclaved for repeated use. Thus, based on the above advantages, 24- or 48-well plates together with a “sandwich plate cover” can be an effective fermentation device for high-throughput screening to identify high-yielding *Streptomyces* strains.

An increase in antibiotic production is mainly realized by mutagenesis of the parental industrial strain [13]. Mutagenesis can cause the conversion of AT to CG in the genome, and has been widely applied for screening of *Streptomyces* mutant strains with enhanced synthesis of metabolites [14]. Methods to increase antibiotic production include random mutagenesis screening, optimization of fermentation medium [15], optimization of culture conditions [16], and genetic engineering strategies [17]. Classical methods to increase microorganism metabolite yield involve inducing random mutations, such as using physical mutagenesis methods, *e.g.*, UV and microwave mutagenesis [18]. UV mutagenesis is considered an easy and convenient method [19], and produces cyclobutene pyrimidine dimers and pyrimidine photoproducts in DNA double strands to cause base mismatches. In addition, it activates some small molecules including riboflavin, tryptophan and porphyrin to generate reactive oxygen species, which damage DNA and cause further gene mutation [20]. Chemical mutagenesis methods include ethyl methanesulfonate (EMS), sodium nitrite, nitrosoguanidine (NTG), and N-methyl-N-nitro-N-nitrosoguanidine (MNNG) [21]. Sodium nitrite can cause oxidative deamination of the bases, turning guanine into xanthine, adenine into hypoxanthine, and cytosine into uracil. The biological effect of hypoxanthine is to cause the conversion of AT to GC and GC to AT in the DNA chain [22-24]. Traditional mutagenesis is stochastic and does not require a clear knowledge of the genetic background of the species, so it is still considered an effective, rapid way to increase antibiotic production [25].

In this work, we established a high-throughput screening method using 24-well plates to screen for *S. fradiae* mutants with enhanced production of tylosin. After comparison of the mycelial morphology and tylosin yield between *S. fradiae* cultured in 24-well plates and shake flasks, the optimal culture time in 24-well plates was established as 120 h. Mutant libraries were generated by UV and/or sodium nitrite mutagenesis, grown in 24-well plates, and tylosin production was measured by UV spectrophotometry. Primary screening identified 98 mutant strains with higher production of tylosin, and these were also evaluated for tylosin yield in conventional shake-flask culture. Finally, two strains with a significantly enhanced yield of tylosin were obtained.

## Materials and Methods

### Strain and Media

*Streptomyces fradiae* SF-3 from Hubei HVSEN Biotechnology Co., Ltd. was used as the wild-type strain.

*S. fradiae* was cultured in Gause’s No. 1 medium containing: 10 g KNO_3_, 0.8 g K_2_HPO_4_·3H_2_O, 0.5 g MgSO_4_·7H_2_O, 0.1 g NaCl, 0.001 g FeSO_4_·7H_2_O, 20 g agar, and 20 g starch per liter (pH 7.2). The seed medium consisted of 6 g corn steep liquor, 5 g soybean cake flour, 5 g yeast extract, 5 g soybean oil, and 3 g CaCO_3_ per liter (pH 7.2). The fermentation medium contained 41.4 g soybean oil, 14 g corn flour, 8 g corn protein flour, 7 g fish meal, 2 g cottonseed flour, 4 g peanut meal, 5 g hot fried soybean cake flour, 0.9 g betaine hydrochloride, 6 mg CoCl_2_·6H_2_O, 4 mg NiSO_4_·6H_2_O, 0.1 g (NH_4_)_2_HPO_4_, 0.3 g MgSO_4_·7H_2_O, and 2 g CaCO_3_ per liter (pH 7.0). The above-mentioned medium was sterilized at 121°C for 20 min prior to use.

### Fermentation in 24-Well Plates

Mature single spores were inoculated into 24-well plates with a pipette tip containing 2 ml seed medium and cultured for 48 h at 30°C, 220 r/min. Then, the culture solution was transferred to 24-well plates at 10% (v/v) containing 1.5 ml fermentation medium for further fermentation at 30°C and 220 r/min for 5 days. The yield of tylosin was determined by absorbance at 290 nm in a UV spectrophotometer.

### Fermentation in Shake Flasks

Mature spores of *S. fradiae* on Gause’s No. 1 plates were washed with sterile normal saline and adjusted to 1×10^7^ CFU using a hemocytometer, and 1 ml spore suspensions were inoculated into 250 ml shake flasks containing 50 ml seed medium for pre-culture for 48 h at 30°C and 220 r/min. The cultured seed medium at an inoculation ratio of 10% (v/v) was transferred to 250 ml shake flasks containing 30 ml fermentation medium for further fermentation for 156 h at 30°C and 220 r/min. Thereafter, the shaker temperature was adjusted to 39°C for continued cultivation for 12 h. The purpose of this step was to facilitate the conversion of component macrocin C to tylosin A to reduce the production of impurity macrocin and increase the production of tylosin A. Tylosin yield in the shake flasks was determined by HPLC.

### Mycelial Morphology

The fermentation broth was diluted with normal saline and a drop of the suspension was spread on a glass slide. Staining was performed for 1 min with a drop of 2% crystal violet solution, followed by a wash with water and air-drying. A 100× oil lens was used to observe mycelial morphology.

### Ultraviolet Mutagenesis of *S. fradiae*

Spores of *S. fradiae* on Gause’s No. 1 plates were washed with sterile normal saline, and filtered using absorbent cotton to remove the mycelium. For UV mutation, spore suspensions were adjusted to 1 × 10^7^ CFU, followed by dilution to 10^-4^ concentration with saline, spread on Gause’s No. 1 plates, and exposed to UV light (20 W) treatment for 5 s, 10 s, 15 s, 20 s, 25 s, 30 s, and 35 s with a 60 cm distance. Spores without UV treatment were used as the control. After treatment, Gause’s No. 1 plates were placed in the dark and grown at 30°C for 15 days to avoid light repair effects [26]. The positive mutation rate and lethality was calculated based on the following equations: Positive mutation rate = (P/M) × 100% [27]; Lethality (%) = (U-T) /U × 100% [28], where M equals the total colony number of the mutant strains, and P equals the colony number of the mutants with higher yield of tylosin than that of the original strain. U equals the total number of colonies in untreated controls; T equals the total number of colonies after mutagenesis. Single colonies of different mutagenesis treatment groups were inoculated into 24-well plates to determine the positive mutation rate. Fifty-two single colonies of mutants treated for 25 s were randomly selected and seeded in microplates for primary screening. The strains with a tylosin yield 10%higher than that of the wild-type strain were inoculated into shake flasks for re-screening.

### Sodium Nitrite Mutagenesis of *S. fradiae* Spore Suspensions

Spore suspensions were prepared as described for UV treatment. One milliliter of single spore solution was mixed with 1 ml of 0.1 M sodium nitrite solution in the same tube, and 2 ml of 1 M acetic acid buffer at pH 4.5 was immediately added, followed by incubation in a water bath at 30°C; treatment was for 10 min, 20 min, 30 min, 40 min, 50 min, or 60 min. The reaction was terminated with addition of 3 ml 0.07 M Na_2_HPO_4_ buffer at pH 8.6. The mixed solution was transferred onto Gause’s No. 1 plates and cultivated at 30°C for 15 days. Mature single spores were inoculated into 24-well plates to calculate lethality and positive mutation rates based on the tylosin yields determined by UV spectrophotometry. Strains with a 10% higher yield than that of the wild-type strain were inoculated into shake flasks and tylosin A yields were determined by HPLC.

### Combined UV and Sodium Nitrite Mutagenesis of *S. fradiae*

A combination treatment by UV and sodium nitrite mutagenesis was used to further improve the mutant yield. Based on single-factor mutagenesis results, wild-type strain spore suspensions were treated with sodium nitrite in a water bath for 20 min at 30°C, followed by exposure to 20 W UV light treatment for 20 s at a distance of 60 cm. After the treatment, spore suspensions were spread on Gause’s No. 1 plates, and incubated at 30°C in the dark for 15 days.

### Determination of Tylosin Yield by UV Spectrophotometry

Fermentation products were centrifuged at room temperature at 2,134 ×*g* for 15 min. Supernatants were diluted in 0.1 M HCl and tylosin content determined by absorbance at 290 nm. The measured absorbance values were converted to the tylosin yield, by comparison with tylosin standard samples at 10, 15, 20, 25, 30 and 35 μg/ml.

### Determination of Tylosin Yield by HPLC

The fermentation broth samples were centrifuged at 2,134 ×*g* for 15 min and the supernatant was collected in a methanol solution and then passed through a 0.22 μm filter to determine the tylosin concentration by HPLC (1260, Agilent, USA). The analysis was performed in a C-18 column (ODS-3, 4.6 × 250 mm, 5 μm). Each sample was injected into the HPLC column in a 20 μl volume and detected at 280 nm. The mobile phase consisted of sodium perchlorate and acetonitrile in a proportion of 60:40 (v/v) [29]. Tylosin A, B, C, and D components were detected at a flow rate of 1.0 ml/min and the temperature of the column was maintained at 30°C.

### Statistical Analysis

Statistical analysis was performed by GraphPad prism 7.0 using the unpaired, two-tailed *t*-test method for significance analysis. A *p*-value of < 0.05 was deemed significantly different, indicated by “*”, while “**” indicated very significantly different (*p* < 0.01).

## Results

### Comparison of the Mycelial Morphology and Tylosin Production in Shake Flasks and 24-Well Plates

**Fig. 1:**
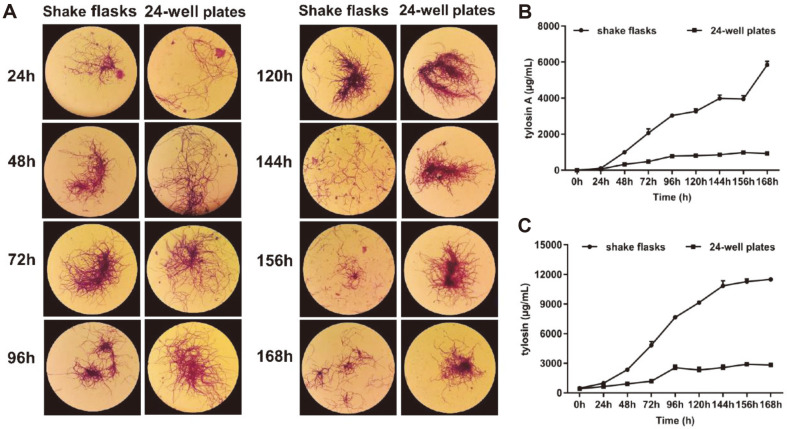
Comparison of the mycelial morphology and tylosin production in shake flasks and 24-well plates. **A**: Mycelia in shake flasks and 24-well plates were sampled every 24 h were observed by microscopy. **B**: Dynamic monitoring of the yields of tylosin A as measured by HPLC obtained with shake flasks or 24-well plates culture. **C**: Dynamic monitoring of tylosin yields after growth in shake flasks or 24-well plates as measured by UV spectrophotometry (absorbance 290 nm). Data are shown as mean ± SD from three independent replicates in B and C.

The relationship between mycelial morphology and productivity is vital for the fermentation production of *Streptomyces*. Therefore, the mycelial morphology of *S. fradiae* in shake flasks and 24-well plates was observed and compared (Fig. 1A). We found no obvious differences in the mycelial morphology in shake flasks and 24-well plates between 24 h to 168 h. At 24 h, the mycelial began to spread, and the mycelial in shake flasks was slightly longer than that in the 24-well plates. The mycelia became thicker and the diffusion was more obvious at 48–72 h, with a good state being maintained until 96 h. At 120 h, mycelia in the shake flasks began to rupture and the color became shallower, while in the 24-well plates agglomeration appeared. After 144 h, mycelia in both the shake flasks and 24-well plates ruptured. Thereafter, the yield of tylosin in shake flasks and 24-well plates at different time points was determined by UV spectrophotometry and HPLC (Figs. 1B and 1C). The results showed that the trend of tylosin production in shake flasks and 24-well plates was the same. Therefore, UV spectrophotometry could be used to determine tylosin yield in 24-well plates. Tylosin was detectable at 48 h, after which tylosin concentration in the shake flasks increased rapidly, reaching the highest value at 168 h. In 24-well plates, the highest values occurred at 96 h, and then remained constant from 120–168 h. Further increase in fermentation time did not increase the tylosin production. For this reason, 120 h was selected as the optimal fermentation time when 24-well plates were used. This was compared with shake flasks fermentation cultures where 168 h was required for optimal yield.

### Optimization of the Medium Filling Volume of 24-Well Plates

**Fig. 2:**
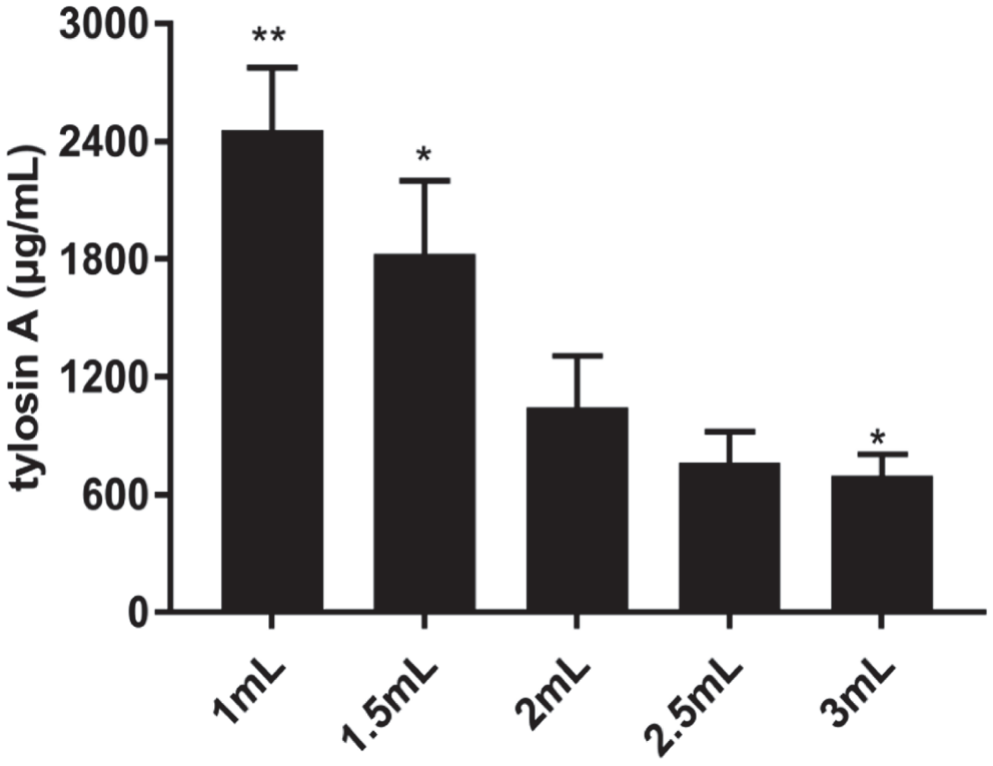
Optimization of the liquid volume of 24-well plates. Tylosin A yield of each medium volume was compared with that from 2 ml medium volume. Data are shown as mean ± SD. “*”, *p* < 0.05; “**” *p* < 0.01.

Since the volume of medium in the 24-well plates is closely related to the dissolved oxygen levels, it was important to determine volume to ensure both successful fermentation and optimal metabolic yield. When the liquid volume was 1 ml, the tylosin yield was determined to be the highest (Fig. 2). In contrast, the tylosin showed the lowest production when the liquid volume was 3 ml (Fig. 2). However, too low a volume is not conducive for downstream determination of fermentation product yield. Thus, we chose 1.5 ml as the optimal volume of fermentation medium in 24-well plates to further screen the *S. fradiae* mutants.

### Screening of High-Yield Strains by UV Mutagenesis

**Fig. 3:**
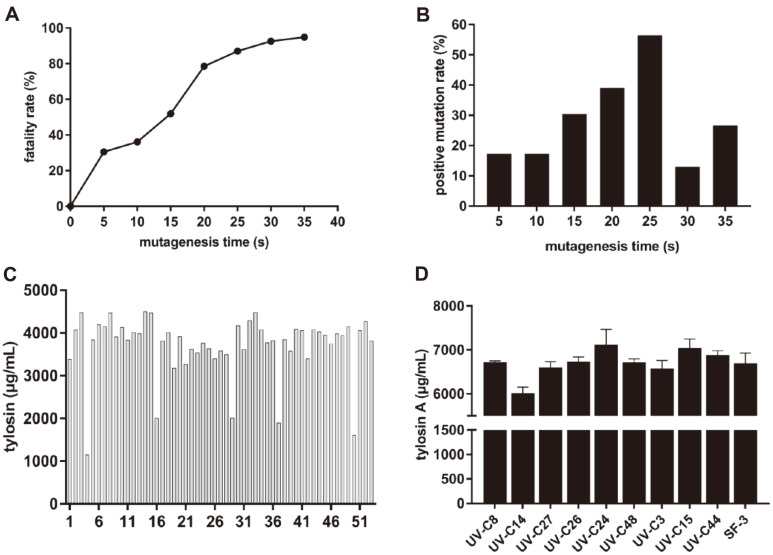
UV mutagenesis for screening of tylosin high-yielding mutants. **A**: Lethality rate of spore suspensions after UV mutagenesis. **B**: Positive mutation rate by UV mutagenesis. **C**: Preliminary screening of high-yield strains detected by UV spectrophotometry after growth in 24-well plates, the last column is wild-type strain SF-3. **D**: Re-screening of high-yield strains detected by HPLC after shake flask culture. In each screening experiment, the wild-type strain SF-3 was used as the control. Data are shown as mean ± SD from three independent replicates.

Spore liquids of SF-3 were treated with UV light at different times. The lethality of UV mutagenesis increased with the prolongation of mutagenesis time from 5 s to 35 s. When the mutagenesis time was 30 s and 35 s, the fatality was 92.66% and 94.91%, respectively (Fig. 3A). At each time point, individual colonies were picked and inoculated into 24-well plates to assess positive mutation rate. With increase of mutagenesis time, positive mutation rates increased in the first 5 s–25 s; the highest rate was at 25 s, and this was lower at 30 s and 35 s (Fig. 3B). Subsequently, 52 single colonies generated by UV mutagenesis for 25 s were inoculated into the 24-well plates for primary screening, and 9 mutants with 10% higher tylosin yields than that of the wild-type strain were obtained (Fig. 3C). These mutants were re-screened in shake flasks for tylosin yields determined by HPLC. The tylosin yield of component A was increased in 6 mutants. The strain with the highest yield of tylosin A was UV-C24, which was 6.4% higher than that of the wild-type strain (Fig. 3D).

### Screening of High-Yield Strains by Sodium Nitrite Mutagenesis

**Fig. 4:**
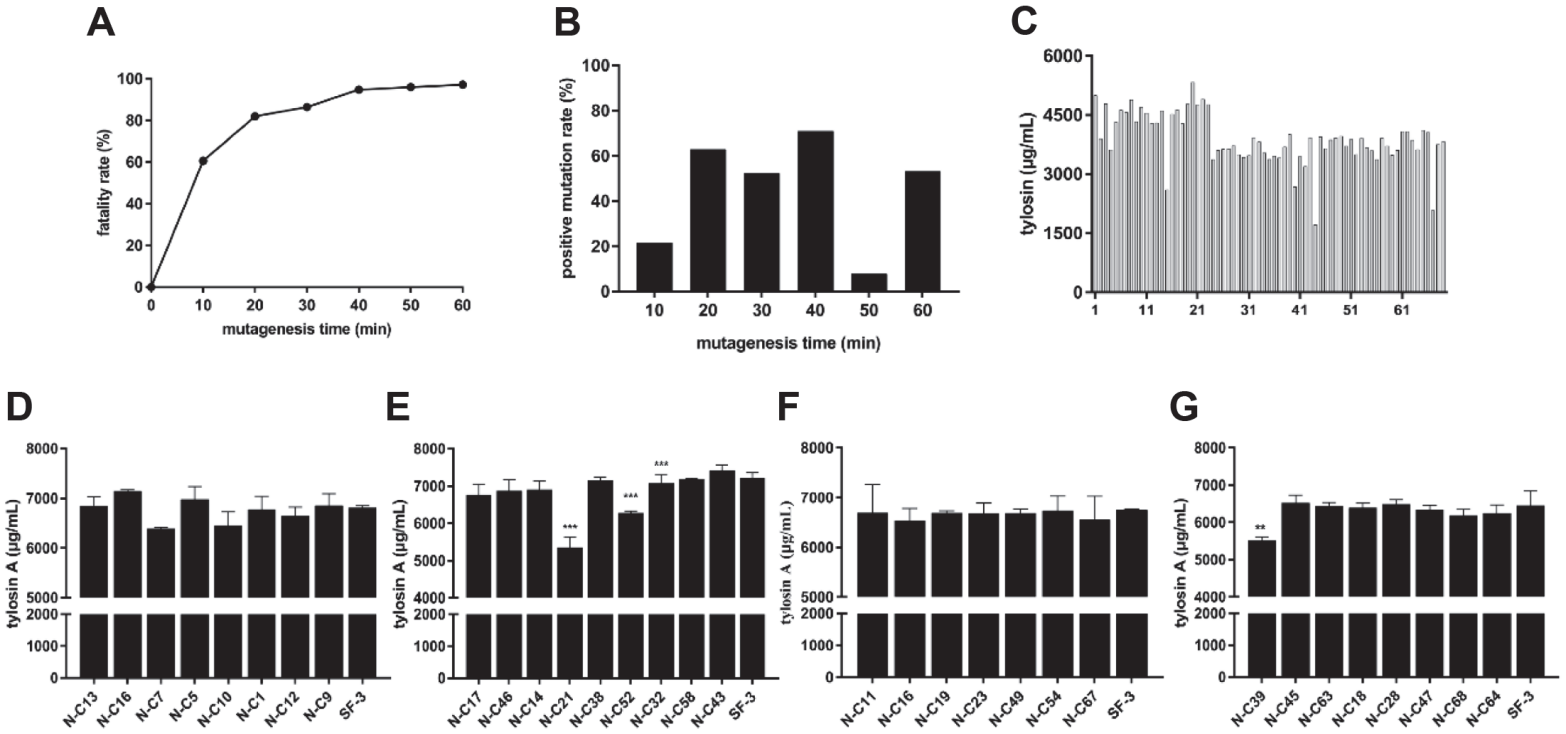
Sodium nitrite mutagenesis for screening of tylosin high-production mutants. **A**: Lethal curve of sodium nitrite mutagenesis. **B**: Positive mutation rate of sodium nitrite mutagenesis. **C**: Preliminary screening of high-yield strains by sodium nitrite in 24-well plates, the last column is wild-type strain SF-3. **D-G**: Rescreening of high-yield strains in shake flasks. In each screening experiment, the wild-type strain SF-3 was used as the control. Data are shown as mean ± SD from three independent replicates.

Sodium nitrite mutagenesis was conducted on the wild-type strain for different times, and the lethality rate and optimal mutation time were determined. The wild-type strain was more sensitive to sodium nitrite, and the lethality of the strains increased with the increase of mutagenesis dose. When the mutagenesis time was 60 min, the lethality rate reached 97.19% (Fig. 4A). The results revealed that the highest positive mutation rate (71.1%) occurred after 40 min treatment (Fig. 4B). Mutants were obtained after 40 min treatment with sodium nitrite, and 68 single colonies were picked into 24-well plates for primary screening and tylosin content determination by UV spectrophotometry. There were 32 mutants which showed higher tylosin yield than the wild-type strain (Fig. 4C). The strains obtained from the primary screening were then inoculated into shake flasks for re-screening, and the concentration of tylosin A components were detected by HPLC. Finally, 7 strains with higher levels of tylosin A component than wild-type strain were obtained (Figs. 4D-4G).

### Screening of High-Yield Strains by Combination of UV and Sodium Nitrite Mutagenesis

**Fig. 5:**
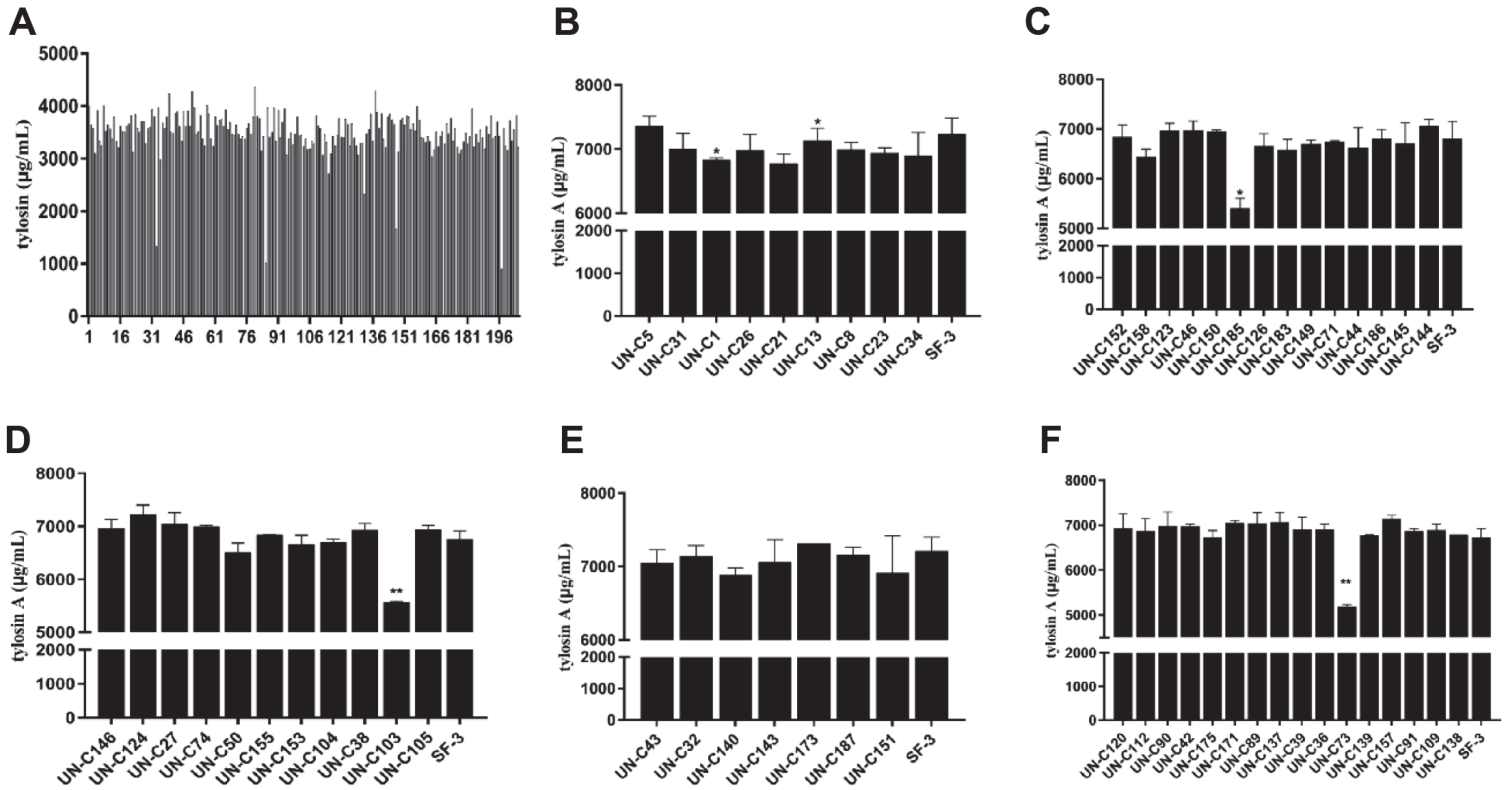
Combination of UV and sodium nitrite mutagenesis for screening of tylosin high-production mutants. **A**: Preliminary screening of mutant strains in 24-well plates, the last column is wild-type strain SF-3. **B-F**: Screening of high-yield strains in shake flasks. In each screening experiment, the wild-type strain SF-3 was used as the control. Data are shown as mean ± SD from three independent replicates.

According to the mutation rate and lethality rate of the above results, the combination of UV treatment for 20 s and sodium nitrite treatment for 20 min was the optimal condition for combined mutagenesis. Mutant libraries generated by combined mutagenesis identified 204 single colonies of interest, and these were inoculated into each well of 24-well plates. Fifty- seven strains with tylosin production 10% higher than the wild-type strain were identified (Fig. 5A). These were inoculated into shake flasks, and the tylosin concentration detected by HPLC, with 29 mutants showing higher tylosin yield. The maximum increase in the yield of tylosin A was 6.9% (Figs. 5B-5F).

### Further Confirmation of Tylosin Yields of the Mutants by Fermentation in Shake Flasks

**Fig. 6:**
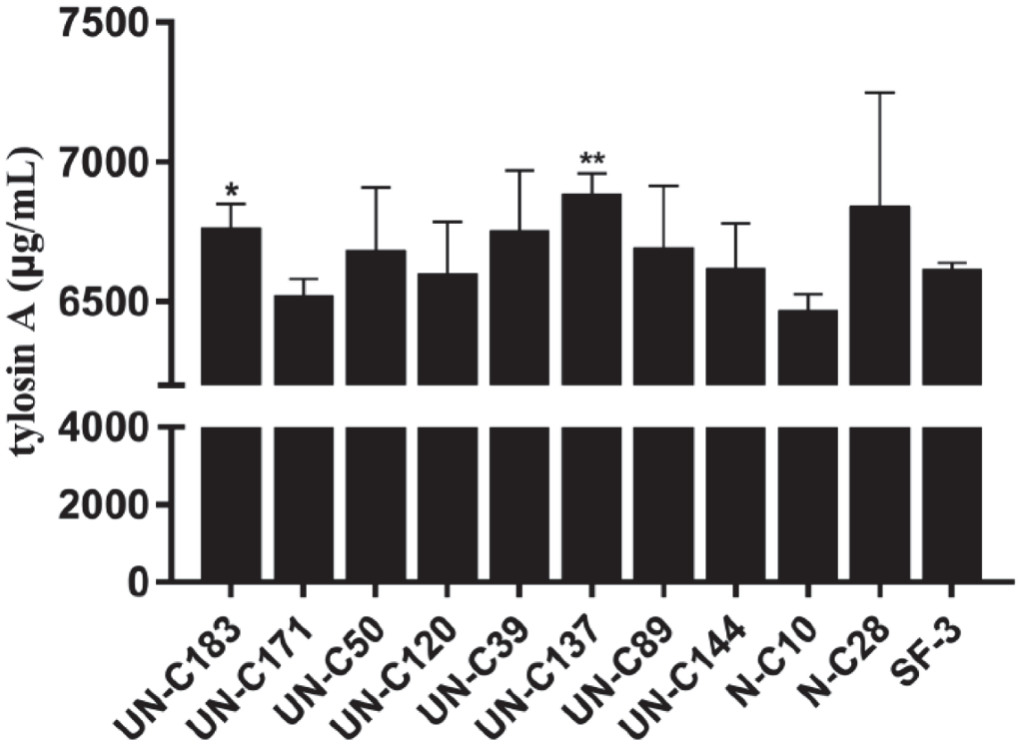
Second-round screening to obtain high yield strains in shake flasks. UN-C183 and UN-C137 showed significantly higher production of tylosin than the wild-type strain SF-3. The wild-type strain SF-3 was used as the control. Data are shown as mean ± SD from three independent replicates. “*” *p* < 0.05; “**” *p* < 0.01.

The strains with the highest yields obtained from the first round of screening in shake flasks were inoculated into shake flasks again for a second round of screening to confirm the yields of tylosin. The tylosin yield of UN-C183 (6767.64 ± 82.43 μg/ml) and UN-C137 (6889.72 ± 70.25 μg/ml) was significantly higher than that of the wild-type strain (6617.99 ± 22.67 μg/ml) (Fig. 6).

## Discussion

### Establishment of a Microculture Technology Screening Method

Tylosin is an important clinical drug with excellent pharmacological effects, but the low yields produced by *S. fradiae* wild-type strains limit industrial production. Therefore, the identification of high-yielding tylosin strains is an important step to increase industrial tylosin yields. Physical and/or chemical mutagenesis are the most convenient methods for random selection of high-yielding strains. Our aim in this study was to establish a high-throughput screening method to identify stable *S. fradiae* mutants with enhanced production of tylosin. Historically, screening has mainly been performed by fermentation in shake flasks, but this is laborious and cumbersome. Hence, we evaluated the effectiveness of screening using 24-well plates. Mycelial morphology and tylosin yields after culture of *S. fradiae* in shake flasks and 24-well plates were compared. Mycelial morphology is crucial for the regulation of the fermentation process, being an important indicator of differentiation and secondary metabolism [30]. In this study, the mycelia in shake flasks and 24-well plates revealed no obvious differences. In addition, variation in the levels of tylosin yields in shake flasks and 24-well plates showed the same trend (Fig. 1). The tylosin content in the 24-well plates did not increase after 96 h and could be associated with the change in mycelia. It may also be due to the increase in viscosity after fermentation to the logarithmic growth phase; the viscosity of the fermentation broth increased, which likely reduces the transfer rate of nutrients and gases [31]. The above results indicated that 24-well plates could be used instead of shake flasks to screen candidate mutant strains. Compared with shake flasks, microplates can shorten screening time, and the process is more efficient. In this study, the culture time of *S. fradiae* was shortened to 120 h in 24-well plates, which was 48 h less than with shake flasks. Recently, it was reported that a high-production mutant of *S. fradiae* showing a 45%increase of neomycin sulfate was obtained after 6 rounds of mutagenesis by ARTP treatment in combination with screening in microplates [32]. In this study, tylosin yields were determined by UV spectrophotometry and HPLC assay. The difference between the two methods of determining the production of tylosin is that UV absorbance measures the total yield of tylosin, the wavelength of 290 nm minimizing interference by pigments and other substances [33, 34]. In contrast, the HPLC assay can measure each tylosin component separately according to the peak areas. Here, we chose to measure the level of tylosin A, the major active component. UV spectrophotometry and HPLC assays in both 24-well plates and shake flasks showed a good correlation, which indicated that detection by the former was a reliable indicator of tylosin yield. Thus, UV spectrophotometry was used to screen for high-yielding mutants after mutagenesis, and these were subsequently rescreened in shake flasks and the yields of tylosin components were then analyzed by HPLC.

### Comparison of Mutagenesis Methods

In this study, three mutagenesis methods were used to modify *S. fradiae*. During the first round of screening, UV induced 17.31% of the strains with a higher yield of tylosin,while sodium nitrite led to 47.06% of the strains with a higher yield of tylosin. In the second round of shake flask screening, 21.9% of the strains with a higher yield of tylosin were obtained by sodium nitrite, and 66.67% of the candidate strains were obtained by UV-induced mutation. These results indicate that the UV mutagenesis strains may be unstable, and sodium nitrite showed higher efficiency. A previous study also reported that sodium nitrite mutation efficiency was higher than that of UV mutagenesis [35]. The effect of combined mutagenesis was better than that of the single mutagenesis, indicated by a greater number of candidate mutants obtained in the first-round screening of 24-well plates and re-screening in shake flasks. In comparison to single mutagenesis, a combination of two different methods has previously been used to identify high-yielding strains of *S. fradiae* [36]. Similar results were obtained in this study.

In the mutagenesis of *S. fradiae*, the dosages and treatment times of the mutagens are closely related to the mutagenesis effect. If the dosage is too low, bacterial repair mechanisms can limit the damage. When the mutagenesis dosage is too high, lethality increases, and positive mutation rate decreases [37]. We assessed different mutagenesis times and doses for optimal mutagenesis. The positive mutation rate of UV mutagenesis at 25 s was the highest, at which time the cell death rate was 87.01%. At the highest positive mutation rate by sodium nitrite treatment (40 min), the lethality rate was 94.79%. Therefore, 25 s and 40 min were the optimal mutagen doses of UV and sodium nitrite, respectively.

### Effect of the Medium Filling Volume on the Yield of Tylosin

Filling volume is also essential during fermentation. Due to the long fermentation period of the strains, part of the medium will evaporate during the process. In addition, too little liquid filling hampers sample testing. Too much liquid can lead to an insufficient oxygen supply, resulting in lower tylosin production. In this work, we found that 1.5 ml was the optimal filling volume with a relatively high yield of tylosin and suitable for detection of fermentation product yield. Therefore, this is a successful method for screening high-yield strains of *S. fradiae*. In another study, to improve the production of epothilone B by *Sorangium cellulosum*, optimum filling volume was also an important factor in achieving a successful culture [38].

### Strains with Higher Yields of Tylosin Were Obtained

After combined mutagenesis, strains cultured in 24-well plates were screened using UV spectrophotometry to determine the yield of tylosin. The mutants with tylosin yield 10% higher than the wild-type strain were inoculated into shake flasks for re-screening, and the tylosin concentrations were analyzed by HPLC. The strains with the highest tylosin yields were inoculated into shake flasks for a second round of screening. Two strains, UN-C183 (6767.64 ± 82.43 μg/ml) and UN-C137 (6889.72 ± 70.25 μg/ml), that stably produced high yields of tylosin were obtained, although the nature of the mutations remains to be determined. The mutagenesis may have resulted in single or multiple gene mutations which may be related to altered metabolism. Previously, in *Saccharopolyspora spinosa*, major changes in carbon metabolism, glycolysis, TCA cycle, and amino acid biosynthesis were found to contribute to enhanced yields of spinosad [39]. In another study, mutation of the biosynthetic site of methoxymalonyl-CoA, a precursor of macrolide polyketide synthases, was found to increase the production of macrolide antibiotics in *S. fradiae* [40]. Expression changes in genes involved in secondary metabolite synthesis gene clusters may also be a reason for an increase in the yield of antibiotics [41]. The increases in tylosin were 2.26% (UN-C183) and 4.11% (UN-C137) in the final two mutants. In a previous study, the yield of tylosin reached 1,500 μg/ml, which was increased by 2.7 ± 0.22-fold by UV mutagenesis compared with the wild-type strain. But the yield from the wild-type strain was low, being only 550 μg/ml [42]. In this study, the tylosin produced by the wild-type strain was 6617.99 ± 22.67 μg/ml, a relatively high level compared with many industrial strains. This may limit the elevation of the production of tylosin by mutagenesis. In a previous study, about 200 mutants of *Streptomyces tsukubaensis* resulting from UV mutagenesis were screened in shake-flask culture, and a tacrolimus-enhanced strain with a 12% increased yield was obtained [43]. ARTP mutagenesis of *S. tsukubaensis* resulted in one mutant with an increase of 11.6% of tacrolimus after two rounds of screening [44]. In these studies, the wild-type yields were low, and therefore it may have been comparatively easy to find many higher-yielding strains. Genetic engineering methods are also good strategies to modify strains to increase antibiotic production. For example, genetic engineering has been used to overexpress the regulatory gene *tylR* of *S. fradiae*, which achieved increased tylosin production by 60–70%, with no effect on bacterial growth or biomass accumulation [45]. Therefore, precise genetic engineering methods, as shown in this work, may be a valuable approach to modify mutants to enable further increases in tylosin yields.

## References

[1] Brahimi-Horn MC, Luo SH, Wang SL, Gau SW, Mou DG (1992). Synthesis of hydrolytic enzymes during production of tylosin by *Streptomyces fradiae*. J. Ind. Microbiol..

[2] Seno ET, Pieper RL, Huber FM (1977). Terminal stages in the biosynthesis of tylosin. Antimicrob. Agents Chemother..

[3] Kim E, Song MC, Kim MS, Beom JY, Lee EY, Kim DM (2016). Characterization of the two methylation steps involved in the biosynthesis of mycinose in tylosin. J. Nat. Prod..

[4] Adrio JL, Demain AL (2006). Genetic improvement of processes yielding microbial products. FEMS Microbiol. Rev..

[5] Teeter JS, Meyerhoff RD (2003). Aerobic degradation of tylosin in cattle, chicken, and swine excreta. Environ. Res..

[6] Kreuzman AJ, Turner JR, Yeh WK (1988). Two distinctive O-methyltransferases catalyzing penultimate and terminal reactions of macrolide antibiotic (tylosin) biosynthesis. Substrate specificity, enzyme inhibition, and kinetic mechanism. J. Biol. Chem..

[7] Huang SL, Hassell TC, Yeh WK (1993). Purification and properties of NADPH-dependent tylosin reductase from *Streptomyces fradiae*. J. Biol. Chem..

[8] Buchs J (2001). Introduction to advantages and problems of shaken cultures. Biochem. Eng. J..

[9] Gao H, Liu M, Zhou X, Liu J, Zhuo Y, Gou Z (2010). Identification of avermectin-high-producing strains by high-throughput screening methods. Appl. Microbiol. Biotechnol..

[10] Renciuk D, Blacque O, Vorlickova M, Spingler B (2013). Crystal structures of B-DNA dodecamer containing the epigenetic modifications 5-hydroxymethylcytosine or 5-methylcytosine. Nucleic Acids Res..

[11] Zeng W, Guo L, Xu S, Chen J, Zhou J (2020). High-throughput screening technology in industrial biotechnology. Trends Biotechnol..

[12] Tan J, Chu J, Hao Y, Guo Y, Zhuang Y, Zhang S (2013). High-throughput system for screening of Cephalosporin C high-yield strain by 48-deep-well microtiter plates. Appl. Biochem. Biotechnol..

[13] Qi H, Lv M, Song K, Wen J (2017). Integration of parallel (13) C-labeling experiments and *in silico* pathway analysis for enhanced production of ascomycin. Biotechnol. Bioeng..

[14] Baltz RH (2001). Genetic methods and strategies for secondary metabolite yield improvement in *actinomycetes*. Antonie Van Leeuwenhoek..

[15] Singh V, Khan M, Khan S, Tripathi CK (2009). Optimization of actinomycin V production by *Streptomyces triostinicus* using artificial neural network and genetic algorithm. Appl. Microbiol. Biotechnol..

[16] Yu Z, Shen X, Wu Y, Yang S, Ju D, Chen S (2019). Enhancement of ascomycin production via a combination of atmospheric and room temperature plasma mutagenesis in *Streptomyces hygroscopicus* and medium optimization. AMB Express.

[17] Fishman SE, Cox K, Larson JL, Reynolds PA, Seno ET, Yeh WK (1987). Cloning genes for the biosynthesis of a macrolide antibiotic. Proc. Natl. Acad. Sci. USA.

[18] Lackmann JW, Bandow JE (2014). Inactivation of microbes and macromolecules by atmospheric-pressure plasma jets. Appl. Microbiol. Biotechnol..

[19] Demain AL, Adrio JL (2008). Strain improvement for production of pharmaceuticals and other microbial metabolites by fermentation. Prog. Drug Res..

[20] Ikehata H, Ono T (2011). The mechanisms of UV mutagenesis. J. Rad. Res..

[21] Baltz RH, Stonesifer J (1985). Adaptive response and enhancement of *N*-methyl-*N*'-nitro-*N*-nitrosoguanidine mutagenesis by chloramphenicol in *Streptomyces fradiae*. J. Bacteriol..

[22] Parekh S, Vinci VA, Strobel RJ (2000). Improvement of microbial strains and fermentation processes. Appl. Microbiol. Biotechnol..

[23] Siddiqi O. H. (1962). Mutagenic action of nitrous acid on *Aspergillus nidulans*. Genet. Res..

[24] Kaudewitz F (1959). Production of bacterial mutants with nitrous acid. Nature.

[25] Sivaramakrishnan R, Incharoensakdi A (2017). Enhancement of lipid production in *Scenedesmus* sp. by UV mutagenesis and hydrogen peroxide treatment. Bioresour. Technol..

[26] Fallahpour N, Adnani S, Rassi H, Asli E (2017). Overproduction of erythromycin by ultraviolet mutagenesis and expression of *ermE* gene in *Saccharopolyspora erythraea*. Assay Drug Dev. Technol..

[27] Wang X, Tian X, Wu Y, Shen X, Yang S, Chen S (2018). Enhanced doxorubicin production by *Streptomyces peucetius* using a combination of classical strain mutation and medium optimization. Prep. Biochem. Biotechnol..

[28] Adams E, Liu L, Dierick K, Guyomard S, Nabet P, Rico S (1998). Neomycin: microbiological assay or liquid chromatography?. J. Pharm. Biomed Anal..

[29] Hamidian K, Amini M, Samadi N (2018). Consistency evaluation between matrix components ratio and microbiological potency of tylosin major components. Daru.

[30] Manteca A, Alvarez R, Salazar N, Yague P, Sanchez J (2008). Mycelium differentiation and antibiotic production in submerged cultures of *Streptomyces coelicolor*. Appl. Environ. Microbiol..

[31] van Dissel D, Claessen D, van Wezel GP (2014). Morphogenesis of *Streptomyces* in submerged cultures. Adv. Appl. Microbiol..

[32] Yu F, Zhang M, Sun J, Wang F, Li X, Liu Y (2022). Improved neomycin sulfate potency in *Streptomyces fradiae* using atmospheric and room temperature plasma (ARTP) mutagenesis and fermentation medium optimization. Microorganisms.

[33] Baltz RH, Seno ET (1981). Properties of *Streptomyces fradiae* mutants blocked in biosynthesis of the macrolide antibiotic tylosin. Antimicrob. Agents Chemother..

[34] Seno ET, Baltz RH (1982). S-Adenosyl-L-methionine: macrocin *O*-methyltransferase activities in a series of *Streptomyces fradiae* mutants that produce different levels of the macrolide antibiotic tylosin. Antimicrob. Agents Chemother..

[35] Ghribi D, Zouari N, Jaoua S (2004). Improvement of bioinsecticides production through mutagenesis of *Bacillus thuringiensis* by u.v. and nitrous acid affecting metabolic pathways and/or delta-endotoxin synthesis. J. Appl. Microbiol..

[36] Zhang W, Liu F, Yang M, Liang Q, Zhang Y, Ai D (2014). Enhanced beta-galactosidase production of *Aspergillus oryzae* mutated by UV and LiCl. Prep. Biochem. Biotechnol..

[37] Zhang K, Mohsin A, Dai Y, Chen Z, Zhuang Y, Chu J (2019). Combinatorial effect of ARTP mutagenesis and ribosome engineering on an industrial strain of *Streptomyces albus* S12 for enhanced biosynthesis of salinomycin. Front. Bioeng. Biotechnol..

[38] Long R, Yang W, Huang G (2020). Optimization of fermentation conditions for the production of epothilone B. Chem. Biol. Drug Des..

[39] Luo Y, Ding X, Xia L, Huang F, Li W, Huang S (2011). Comparative proteomic analysis of *Saccharopolyspora spinosa* SP06081 and PR2 strains reveals the differentially expressed proteins correlated with the increase of spinosad yield. Proteome Sci..

[40] Rodriguez E, Ward S, Fu H, Revill WP, McDaniel R, Katz L (2004). Engineered biosynthesis of 16-membered macrolides that require methoxymalonyl-ACP precursors in *Streptomyces fradiae*. Appl. Microbiol. Biotechnol..

[41] Huang K, Zhang B, Chen Y, Liu ZQ, Zheng YG (2020). Comparative transcriptome analysis of *Streptomyces nodosus* mutant with a high-yield amphotericin B. Front. Bioeng. Biotechnol..

[42] Khaliq S, Akhtar K, Afzal Ghauri M, Iqbal R, Mukhtar Khalid A, Muddassar M (2009). Change in colony morphology and kinetics of tylosin production after UV and gamma irradiation mutagenesis of *Streptomyces fradiae* NRRL-2702. Microbiolog. Res..

[43] Yan L, Zhang Z, Zhang Y, Yang H, Qiu G, Wang D (2021). Improvement of tacrolimus production in *Streptomyces tsukubaensis* by mutagenesis and optimization of fermentation medium using Plackett-Burman design combined with response surface methodology. Biotechnol. Lett..

[44] Ye L, Ye R, Hu F, Wang G (2021). Combination of atmospheric and room temperature plasma (ARTP) mutagenesis, genome shuffling and dimethyl sulfoxide (DMSO) feeding to improve FK506 production in *Streptomyces tsukubaensis*. Biotechnol. Lett..

[45] Stratigopoulos G, Bate N, Cundliffe E (2004). Positive control of tylosin biosynthesis: pivotal role of TylR. Mol. Microbiol..

